# Association Between Lactates, Blood Glucose, and Systemic Oxygen Delivery in Children After Cardiopulmonary Bypass

**DOI:** 10.3389/fped.2020.00332

**Published:** 2020-06-23

**Authors:** Philippe Klee, Peter C. Rimensberger, Oliver Karam

**Affiliations:** ^1^Pediatric Endocrine and Diabetes Unit, Department of Pediatrics, Gynecology and Obstetrics, University Hospitals of Geneva, Geneva, Switzerland; ^2^Diabetes Center of the Faculty of Medicine, University of Geneva, Geneva, Switzerland; ^3^Service of Neonatology and Pediatric Intensive Care, Department of Pediatrics, Gynecology and Obstetrics, University Hospitals of Geneva, Geneva, Switzerland; ^4^Division of Pediatric Critical Care Medicine, Children's Hospital of Richmond at VCU, Richmond, VA, United States

**Keywords:** lactic acidosis, hyperglycemia, oximetry, critical illness, cardiac surgical procedures, systemic oxygen delivery

## Abstract

**Objective:** Lactate is often used as a surrogate marker of inappropriate oxygen delivery. It has been shown that hyperlactatemia is associated with worse clinical outcome in children after cardiac surgery. The purpose of this study is to evaluate the association of hyperlactatemia, low systemic oxygen delivery, and hyperglycemia, in children admitted to the pediatric critical care unit after cardiopulmonary bypass.

**Design:** Secondary analysis of an observational cohort study.

**Setting:** Tertiary pediatric critical care unit (PICU).

**Patients:** Ninety-three patients, aged 6 months to 16 years, undergoing cardiac surgery with cardiopulmonary bypass.

**Interventions:** None.

**Measurements and Main Results:** Metabolic tests (blood glucose, lactate, lactate/pyruvate ratio, and ketones) and oxygen extraction (SaO_2_-SvO_2_) were performed before anesthesia, at the end of cardiopulmonary bypass, at PICU admission, and at 4 and 12 h after PICU admission. Four hours after PICU admission, 62% of the patients had hyperlactatemia (>2 mmol/L), of whom 55% had normal oxygen extraction (SaO_2_-SvO_2_ < 30%). There was no correlation between lactate and oxygen extraction (R = −0.09, *p* = 0.41) but there was a moderate correlation between lactate and blood glucose (R = 0.55, *p* < 0.001). Using a logistic regression model, hyperlactatemia at 4 h after PICU admission was independently associated with hyperglycemia (*p* = 0.007) and lactate/pyruvate ratio (*p* = 0.007) at the same timepoint, as well as with lactate at PICU admission (*p* = 0.002), but not with weight (*p* = 0.45), severity of the cardiac lesion (*p* = 0.85), duration of bypass (*p* = 0.16), or oxygen extraction, as evaluated by SaO_2_-SvO_2_ (*p* = 0.54). At 12 h after PICU admission, there was a very week correlation between lactate and blood glucose (R = 0.27, *p* = 0.007), but none between lactate and oxygen extraction (R = 0.13, *p* = 0.20).

**Conclusion:** In children after cardiopulmonary bypass, lactates are not correlated with higher oxygen extraction, but are correlated with hyperglycemia, at both 4 and 12 h after PICU admission. Future research is warranted to better define this relationship.

## Introduction

Hyperlactatemia after cardiopulmonary bypass (CBP) in children is a frequent finding (1, 2) and has consistently been identified to be predictive of adverse events during the early postoperative period (3–5). Often used as a surrogate marker of a low cardiac output, high blood lactate levels can result from tissue hypoperfusion (type A hyperlactatemia) (6), but may also occur in patient with a normal tissue perfusion (type B hyperlactatemia) (7). In those situations, hyperlactatemia may be related to hyperglycemia (8), increased use of red blood cell transfusions (9) or decreased lactate clearance (10). In is thought that type A hyperlactatemia is more prevalent at pediatric intensive care unit (PICU) admission, whereas type B hyperlactatemia may play a more important role after admission to PICU (11). Importantly, the outcome related to hyperlactatemia may be dependent on the underlying cause. Thus, especially hyperlactatemia associated with tissue hypoxia is a major predictor of mortality (11), and differentiating the origin of increased lactates is crucial in the care of children after CPB.

The purpose of this study was to describe relationship between hyperlactatemia, hyperglycemia and low systemic oxygen delivery (assessed by oxygen extraction) in children aged 6 months to 16 years undergoing cardiac surgery and CPB. In this secondary analysis of a prospective observational study (12), we focused on patients presenting with hyperlactatemia, 4 and 12 h after PICU admission, in an attempt to describe risk factors for hyperlactatemia, as well as the origin of lactates, at crucial timepoints where identification of impaired systemic oxygen delivery is important and where a significant percentage of patients presents with hyperglycemia.

## Materials and Methods

### Study Design

This is a *post-hoc* secondary analysis of a prospective observational single-center study involving 93 children consecutively admitted to the PICU of the University Hospital of Geneva, Switzerland. The trial was approved by the local ethical committee. A detailed description of the methods was previously reported (12).

### Patients

Patients 6 months to 16 years old undergoing cardiac surgery with cardiopulmonary bypass (CPB) were eligible for inclusion. We excluded patients undergoing simple repair of isolated ventricular or atrial septal defects (as our goal was to enroll a population at risk of low cardiac output syndrome), as well as patients with diabetes and patients with sepsis, that is, patients presenting with a temperature >38.0°C or laboratory signs of inflammation prior to surgery. Baseline demographic and clinical characteristics were recorded. For the evaluation of the surgical risk, the score on the Risk Adjustment in Congenital Heart Surgery-1 (RACHS-1) score (13) (ranging from 1 to 6, with higher scores indicating greater risk) and the Pediatric Index of Mortality (PIM) 2 (14) (calculating the probability of death in percentage) were assessed. Written informed consent was obtained from each patient or legal surrogate before the surgical procedure.

### Cardiopulmonary Bypass

All patients underwent normo-thermic CPB with alpha-stat management and with continuous and modified ultrafiltration. Methylprednisolone (30 mg/kg) was administered to all patients before the procedure.

### Blood Samples

Blood samples were collected at the following fixed intervals: before anesthesia, at the end of CPB, at PICU admission, and at 4 and 12 h after PICU admission. Arterial blood was sampled at these interval times for pH, glucose, lactate, *C*-peptide, ketones, pyruvate, cortisol, N-terminal pro-brain natriuretic peptide, troponin I, and arterial saturation of oxygen (SaO_2_), and central venous blood (internal jugular) for central venous saturation of oxygen (SvO_2_). Whole blood ketones were measured using a semi-quantitative method, measuring acetoacetic acid and acetone by a colorimetric reaction (Roche Diagnostics, Mannheim, Germany; ref 01 0126187190). Blood glucose levels and oxygen saturations (both SaO_2_ and SvO_2_) were exclusively measured by blood gas analyzers (Radiometer ABL 800 flex; Radiometer, Copenhagen, Denmark) located within the operating room or the PICU. Oxygen extraction was defined as the difference between SaO_2_ and SvO_2_. Hyperglycemia was defined as blood glucose >11 mmol/L (200 mg/dL) and hyperlactatemia as lactate >2 mmol/L (18 mg/dL).

### Glucose Control

Aspart (Novorapid; Novo Nordisk Pharma, Küsnacht, Switzerland) insulin diluted in saline was administered IV when blood glucose levels exceeded 12 mmol/L (216 mg/dL), at an initial rate of 0.05 UI/kg/h and adapted according to a detailed paper protocol available to all physicians. The treatment was discontinued when blood glucose levels fell below 10 mmol/L (180 mg/dL). As from PICU admission, all patients received a minimum glucose dose of 4 mg/kg/min, whereas no glucose was administered during surgery, unless hypoglycemia <3.3 mmol/L (<60 mg/dL) was identified. No patient included into this study received enteral nutrition during the period of observation.

### Statistical Analysis

Data are presented as medians and interquartile ranges, and as percentages for categorical variables. Some continuous variables (lactate, blood glucose, lactate/pyruvate ratio) were further categorized, using the median as the cutoff. Continuous variables were analyzed with non-parametric Mann-Whitney test for independent samples. Categorical variables were analyzed with the chi-square test. Correlations were assessed with the Spearman test. Based on the *R* value, the correlation was considered very strong (*R* above 0.9), strong (*R* between 0.7 and 0.9), moderate (*R* between 0.5 and 0.7), weak (*R* between 0.3 and 0.5), very weak (*R* between 0.1 and 0.3), or no linear relationship (*R* below 0.1) (15).

Multivariate regression models were used to test the relationship between lactate (as a continuous and a categorized variable) and oxygen extraction at 4 and 12 h after PICU admission, adjusting for weight, severity of the cardiac lesion as defined by the RACHS-1 score, duration of CPB, lactate at PICU admission, as well as blood glucose, lactate/pyruvate ratio, and ketones 4 h after PICU admission.

### Sample Size

As there was no preliminary data to allow for sample size calculation, we decided a priori to enroll up to 100 patients over a 2-year period, whichever came first.

## Results

A total of 96 patients were consecutively included between April 2012 and May 2014. Three patients were excluded a posteriori from this secondary analysis as due to missing data. The types of pathologies were as follows: Valvular disease 48 (52%), Tetralogy of Fallot 23 (25%), Transposition of great arteries 4 (4%), Total anomalous pulmonary venous return 2 (2%), Truncus arteriosus 1 (1%), and other complex diagnoses 15 (16%). All patients had aortic cross-clamp (median duration 44 min, IQR 27; 60). The baseline demographics are shown in Table 1.

**Table 1:** Demographics and biological characteristics, according to lactate values 4 h after PICU admission.

**Variables**	**All patients** ***n* = 93**	**Lactate, 4 h after PICU admission**		
		**≤2 mmol/L**	**>2 mmol/L**	
		***n* = 35**	***n* = 58**	***p-*value**
Gender [% male]	54 (58%)	22 (63%)	32 (54%)	0.37
Age [months]	85 (23; 154)	54 (20; 132)	97 (23; 167)	**0.04**
Weight [kg]	17.1 (10.3; 34.5)	14.6 (8.2; 25.0)	19.9 (10.6; 36.8)	0.10
Weight <5th percentile	57 (61%)	21 (60%)	36 (62%)	0.65
RACHS-1 score ≥3	73 (78%)	27 (77%)	46 (79%)	0.69
PIM2 score	2.0 (1.3; 3.8)	1.9 (1.3; 5.0)	2.0 (1.3; 3.6)	0.51
Duration of CPB [min]	69 (58; 89)	68 (53; 85)	68 (58; 89)	0.48
**AT PICU ADMISSION**				
Glucose [mmol/L]	8.8 (6.3; 11.1)	6.5 (5.4; 9.0)	9.2 (7.0; 12.6)	**<0.001**
Lactate [mmol/L]	1.8 (1.4; 2.7)	1.4 (1.0; 1.8)	2.3 (1.7; 2.8)	**<0.001**
Lactate/pyruvate	18.4 (14.5; 21.6)	16.6 (13.5; 19.3)	19.0 (15.0; 22.7)	**0.01**
SaO_2_-SvO_2_ [%]	25 (19; 32)	22 (18; 31)	26 (20; 32)	0.44
Ketosis	30 (32%)	17 (49%)	13 (22%)	**0.007**
**FOUR HOURS AFTER PICU ADMISSION**				
Glucose [mmol/L]	12.2 (9.0; 15.3)	8.9 (6.9; 12.3)	13.6 (11.4; 17.8)	**<0.001**
Glucose >11.1 mmol/L	56 (60%)	10 (29%)	46 (79%)	**<0.001**
Lactate [mmol/L]	2.4 (1.4; 3.4)	1.3 (1.1; 1.5)	2.9 (2.4; 4.2)	**<0.001**
Lactate/pyruvate	17.1 (14.7; 20.3)	16.1 (14.0; 17.3)	18.3 (15.0; 22.2)	**0.008**
SaO_2_-SvO_2_ [%]	29 (24; 33)	30 (26; 34)	29 (24; 33)	0.59
SaO_2_-SvO_2_ >30%	44 (47%)	18 (51%)	26 (45%)	0.54
Ketosis	27 (29%)	15 (43%)	12 (21%)	**0.02**
**TWELVE HOURS AFTER PICU ADMISSION**				
Glucose [mmol/L]	11.0 (8.8; 13.7)	10.6 (8.9; 13.9)	11.3 (8.8; 13.7)	**0.02**
Glucose >11.1 mmol/L	48 (52%)	16 (46%)	31 (53%)	0.38
Lactate [mmol/L]	1.5 (1.1; 2.8)	1.2 (0.9; 1.6)	1.8 (1.3; 2.6)	**<0.001**
Lactate/pyruvate	14.5 (12.0; 17.2)	13.9 (12.0:15.5)	14.8 (12.0; 17.2)	**<0.001**
SaO_2_-SvO_2_ [%]	33 (26; 38)	32 (27; 37)	34 (24; 39)	0.53
SaO_2_-SvO_2_ >30%	54 (58%)	18 (51%)	36 (62%)	0.54
Ketosis	22 (24%)	9 (26%)	14 (24%)	0.49
**CLINICAL OUTCOMES**				
Length of mechanical ventilation [hours]	9 (3; 31)	10 (4; 22)	9 (3; 46)	0.78
PICU mortality	3 (3%)	0 (0%)	3 (5%)	0.18
Hospital mortality	3 (3%)	0 (0%)	3 (5%)	0.18

*RACHS-1 score, Risk Adjustment in Congenital Heart Surgery-1; PIM2, Pediatric Index of Mortality 2 score; CPB, Cardiopulmonary Bypass; PICU, Pediatric Intensive Care Unit; SaO_2_, Arterial saturation of oxygen; SvO_2_, Central venous saturation of oxygen. P-values in bold identify statistical significance*.

### Association Between Lactates, Oxygen Extraction, and Blood Glucose, at 4 h

**Figure 1:**
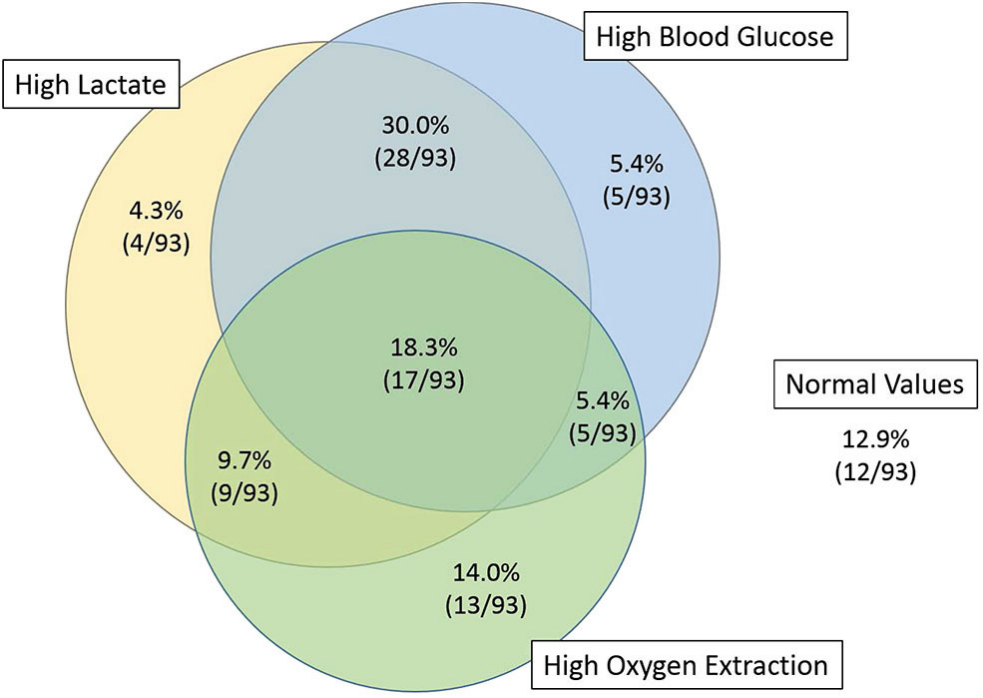
Venn diagram of the patients with high lactates, high blood glucose, and high oxygen extraction, 4 h after PICU admission. Fifty-five percent of the patients with high lactate (32/58) had normal oxygen extraction.

**Figure 2:**
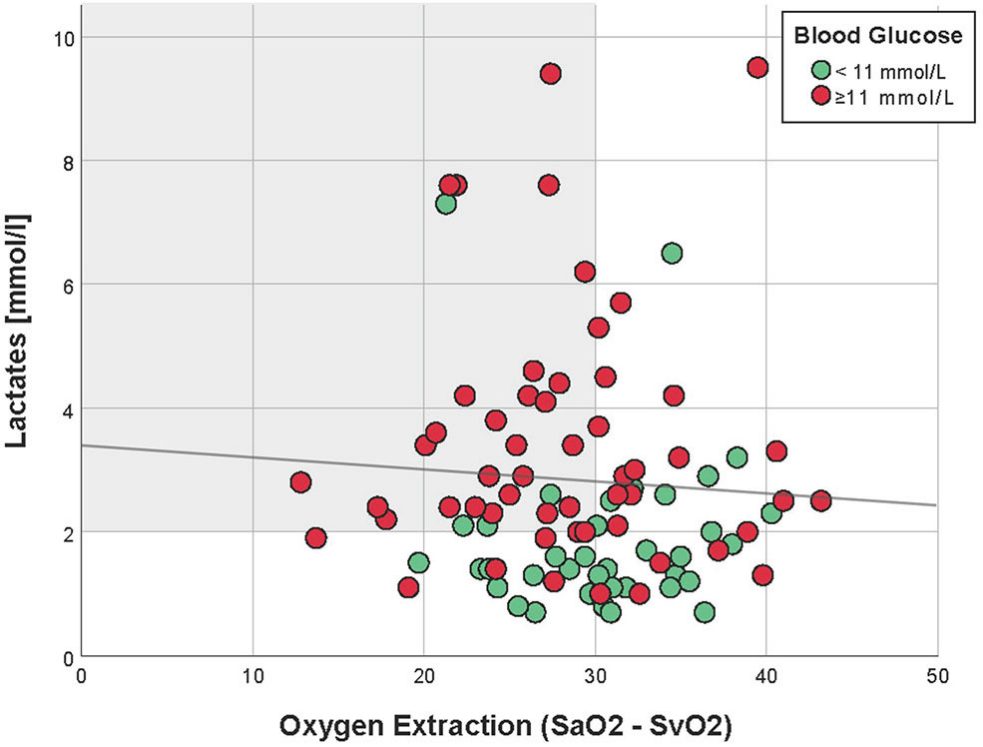
Correlation between oxygen extraction and lactates, 4 h after PICU admission (Spearman R = −0.09, *p* = 0.41). Patients with hyperglycemia (≥11 mmol/L) are in red, whereas those with normal blood glucose are in green. The light gray zone represents the area of normal oxygen extraction but high lactates.

Four hours after PICU admission, 62% (58/93) of the patients had a lactate >2 mmol/L, of whom 45% (26/58) had increased oxygen extraction, as defined by SaO_2_-SvO_2_ >30% (Table 1 and Figure 1). There was no correlation between lactate and oxygen extraction (R = −0.09, *p* = 0.41, Figure 2). However, there was a moderate association between lactate and blood glucose (R = 0.55, *p* < 0.001).

Patients with lactates >2 mmol/L 4 h after PICU admission, were older, had higher glucose, lactate/pyruvate ratios, and less frequent ketosis, both at PICU admission and 4 h after PICU admission (Table 1). Patients with hyperglycemia 4 h after PICU admission had higher lactates [2.9 mmol/L (IQR 2.1; 4.2)] compared to patients with normal glucose [1.5 mmol/L (IQR 1.1; 2.3), *p* < 0.001].

In patients with hyperlactatemia and normal blood glucose at 4 h after PICU admission, oxygen extraction seemed to be higher in the patients with high lactate/pyruvate ratios, reflecting a probable hypoxic origin of lactates. Conversely, in hyperglycemic patients, no statistically significant difference in lactate/pyruvate ratio could be found, reflecting a mixed (metabolic and hypoxic) origin of lactates.

Using a logistic regression model, hyperlactatemia at 4 h after PICU admission was independently associated with hyperglycemia (*p* = 0.007), lactate at PICU admission (*p* = 0.002), and lactate/pyruvate ratio (*p* = 0.007), but not with weight (*p* = 0.45), severity of the cardiac lesion (*p* = 0.85), duration of bypass (*p* = 0.16), or oxygen extraction, as evaluated by SaO_2_-SvO_2_ (*p* = 0.54, Table 2).

**Table 2:** Factors associated with hyperlactatemia (lactate >2 mmol/L), 4 h after PICU admission.

**Variable**	**Adjusted odds ratio**	**95% confidence interval**	***p*-value**
Weight	1.02	0.97–1.06	0.45
RACHS score	1.15	0.28–4.59	0.85
Duration of cardiopulmonary bypass	1.01	0.99–1.04	0.16
Lactate, at PICU admission (per mmol/L)	4.63	1.77–12.12	**0.002**
Glucose, 4 h after PICU admission (per mmol/L)	1.16	1.04–1.28	**0.007**
SaO_2_-SvO_2_, 4 h after PICU admission	1.03	0.94–1.12	0.54
Ketosis, 4 h after PICU admission	0.34	0.08–1.58	0.17
Lactate/pyruvate, 4 h after PICU admission	1.29	1.07–1.55	**0.007**

*RACHS-1 score, Risk Adjustment for Congenital Heart Surgery score; PICU, Pediatric Intensive Care Unit. P-values in bold identify statistical significance*.

### Association Between Lactates, Oxygen Extraction, and Blood Glucose, at 12 h

Twelve hours after PICU admission, 31% (29/93) of the patients had a lactate >2 mmol/L, of whom 72% (21/29) had increased oxygen extraction, as defined by SaO_2_-SvO_2_ >30% (Venn diagram available in the Supplemental Online Data). Glucose, lactate/pyruvate ratio, and ketosis are described in Table 1. There was no correlation between lactate and oxygen extraction (R = 0.13, *p* = 0.20. However, there was a very weak association between lactate and blood glucose (R = 0.27, *p* = 0.007) (see Supplemental Online Data for additional graphs and regression models).

### Clinical Outcomes

At 12 h, only one patient had a lactate above 5 mmol/L and 17 (18%) had an oxygen extraction >40%. As shown in Table 1, apart from the lactate levels at 12 h, there were no statistically significant differences in outcome for the patients. Overall, the length of mechanical ventilation was 9 h (IQR 3; 30). Three patients (3.1%) did not survive (case descriptions in Supplemental Data).

## Discussion

In this study, we have analyzed the relation between hyperlactatemia, low systemic oxygen delivery, and hyperglycemia, 4 and 12 h after PICU admission. Our results indicate that, at 4 h after PICU admission, a large proportion of patients are hyperlactatemic, hyperglycemic, and/or have high oxygen extraction. At that timepoint, we found that lactates were not independently associated with higher oxygen extraction, but were independently associated with hyperglycemia. Similarly, at 12 h, lactates were not correlated with higher oxygen extraction, but were correlated with hyperglycemia. However, at that timepoint, there was no independent association between hyperglycemia and hyperlactatemia.

There is probably a mixed origin of lactates in patients after cardiac surgery (6). As hyperlactatemic patients, 4 h after PICU admission, had a lower prevalence of ketosis, it is possible some high lactate levels might be resulting from increased glucose utilization via glycolysis and subsequent conversion of pyruvate into lactates (8, 16), in the absence of tissue hypoxia. Although it has been previously shown that a lactate/pyruvate ratio is higher during anaerobic production of lactates (17), we were not able to better differentiate the origin of lactates, despite performing a subgroup analysis of patients with hyperlactatemia, normal blood glucose values, and high lactate/pyruvate ratios. One might hypothesize that the relation between oxygen extraction and the lactate/pyruvate ratio is blunted in hyperglycemic patients, where lactates presumably come both from anaerobic metabolism and increased glycolysis, with subsequent increase in both lactate and pyruvate, and leading to an unpredictable ratio.

Although some clinicians use lactate as surrogate marker of a low cardiac output, the clinical impact of hyperlactatemia is still unclear. Whereas, some authors have shown an association with adverse events during the early postoperative period (3–5), our resuls, as well of those of Jackman et al., did not find an association between lactates and poor clinical outcome (18). This might be due to different case-mix and different incidences of low cardiac output syndromes, between the various studies.

The strengths of this study are the prospective analysis of the metabolic constellation of children after CPB and the detailed analysis of specific patient groups. It highlights the lack of correlation between hyperlactatemia and increased oxygen extraction, 4 and 12 h after PICU admission.

We acknowledge several limitations. First, the total number of patients, as well as of patients with a very high oxygen extraction included in this study may have been insufficient to find correlations between hyperlactatemia and other metabolic markers, especially when subgroup analyses were performed. Therefore, we could not hypothesize on the origin of hyperlactatemia (e.g., in hyperglycemic patients with normal oxygen extraction or normoglycemic patients with increased oxygen extraction). Second, this study being observational, we could not describe the potential effect of insulin on the prevalence of hyperlactatemia and on the correlation between hyperlactatemia and hyperglycemia and/or oxygen extraction. It is possible that treating hyperglycemic patients with insulin may modify the prevalence of the different mechanisms leading to lactate production. Third, our design was not able to assess the impact of ultrafiltration and steroids during bypass. Fourth, only a small minority of our patients had signs of low cardiac output at 12 h, as only one patient had a lactate >5 mmol/L. Finally, the mean age of our patient population is ~7 years. The metabolism of newborns being different, our study's results may not be applicable in a younger patient population.

Since the identification of shock is crucial and probably a major determinant of clinical outcomes, this study lays the ground for future larger observational studies, to better identify biomarkers (such as lactates and SaO_2_-SvO_2_) and describe their performance to predict low cardiac output syndrome. These studies should also allow for detailed analysis of metabolic subgroups, as well as to evaluate the effect of early insulin on the prevalence and origin of lactates. These studies should allow for a longitudinal follow-up of lactate levels, in order to identify hyperlactatemia but also lactate change over time—a parameter that also has a negative impact on clinical outcome (4, 19, 20). Finally, as lactate levels are influenced by the administration of lactate from packed red blood cell transfusions (10), the effect of transfusions should also be evaluated.

## Conclusion

In our series of children undergoing cardiac surgery, for which we measured lactate, glycemia, and oxygen extraction at PICU admission, and 4 and 12 h later, our results suggest half of the patients with hyperlactatemia at 4 h after PICU admission had normal oxygen extraction. At the 4-h timepoint, lactates were not independently associated with higher oxygen extraction, but were independently associated with hyperglycemia. Similarly, at 12 h, lactates were not correlated with higher oxygen extraction, but were correlated with hyperglycemia. However, at the 12-h timepoint, there was no independent association between hyperglycemia and hyperlactatemia. Further studies are needed to identify reliable markers of shock in children after cardiac surgery.

## Data Availability Statement

The raw data supporting the conclusions of this article will be made available by the authors, without undue reservation.

## Ethics Statement

The studies involving human participants were reviewed and approved by Swiss Ethics Committees on research involving humans. Written informed consent to participate in this study was provided by the participants' legal guardian/next of kin.

## Author Contributions

PK wrote the research project, wrote the manuscript, and analyzed data. PR wrote the research project and reviewed the manuscript. OK reviewed the manuscript and analyzed data. PK and OK are the guarantors of this work, had full access to all the data, and take responsibility for the integrity of the data. All authors contributed to the article and approved the submitted version.

## Conflict of Interest

The authors declare that the research was conducted in the absence of any commercial or financial relationships that could be construed as a potential conflict of interest.
