# Supplementary material for: Association Between Lactates, Blood Glucose, and Systemic Oxygen Delivery in Children After Cardiopulmonary Bypass
Source: Front Pediatr. 2020 Jun 23;8:332. doi: 10.3389/fped.2020.00332 (PMC7325592; doi:10.3389/fped.2020.00332)
Supplement: Supplementary file 1 [file Data_Sheet_1.PDF]

Association between Lactates, Blood Glucose, and Systemic Oxygen Delivery in Children after Cardiopulmonary Bypass

Philippe Klee, Peter Christoph Rimensberger, and Oliver Karam

Supplemental Online Data

1) Proportion of patients with high lactates, high blood glucose, high oxygen extraction, at 12 hours after PICU admission

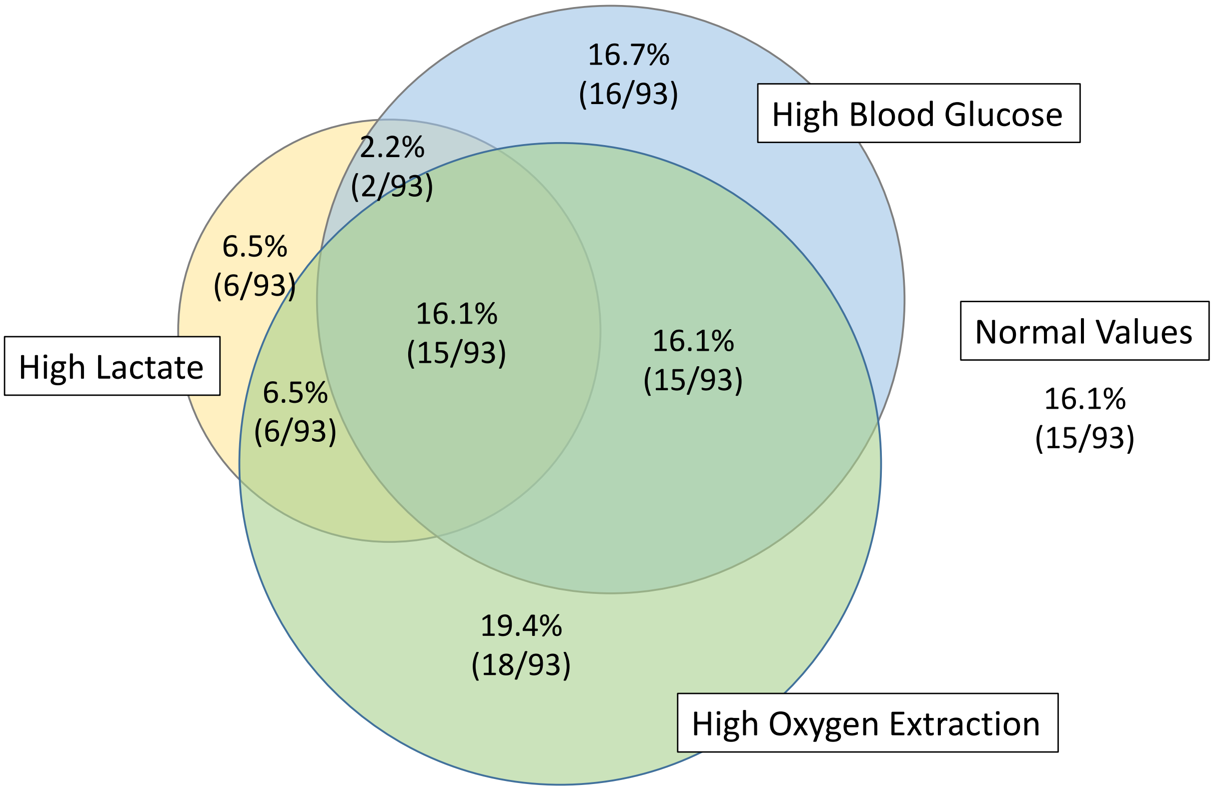

Figure S1: Venn diagram of the proportion of patients with high lactates, high blood glucose, high oxygen extraction, at 12 hours after PICU admission

2) Association between Oxygen Extraction (SaO2-SvO2) and Lactates, at 12 hours

2A. Correlation between continuous variables:

Spearman correlation between Oxygen Extraction and Lactates: R=0.13, p=0.20

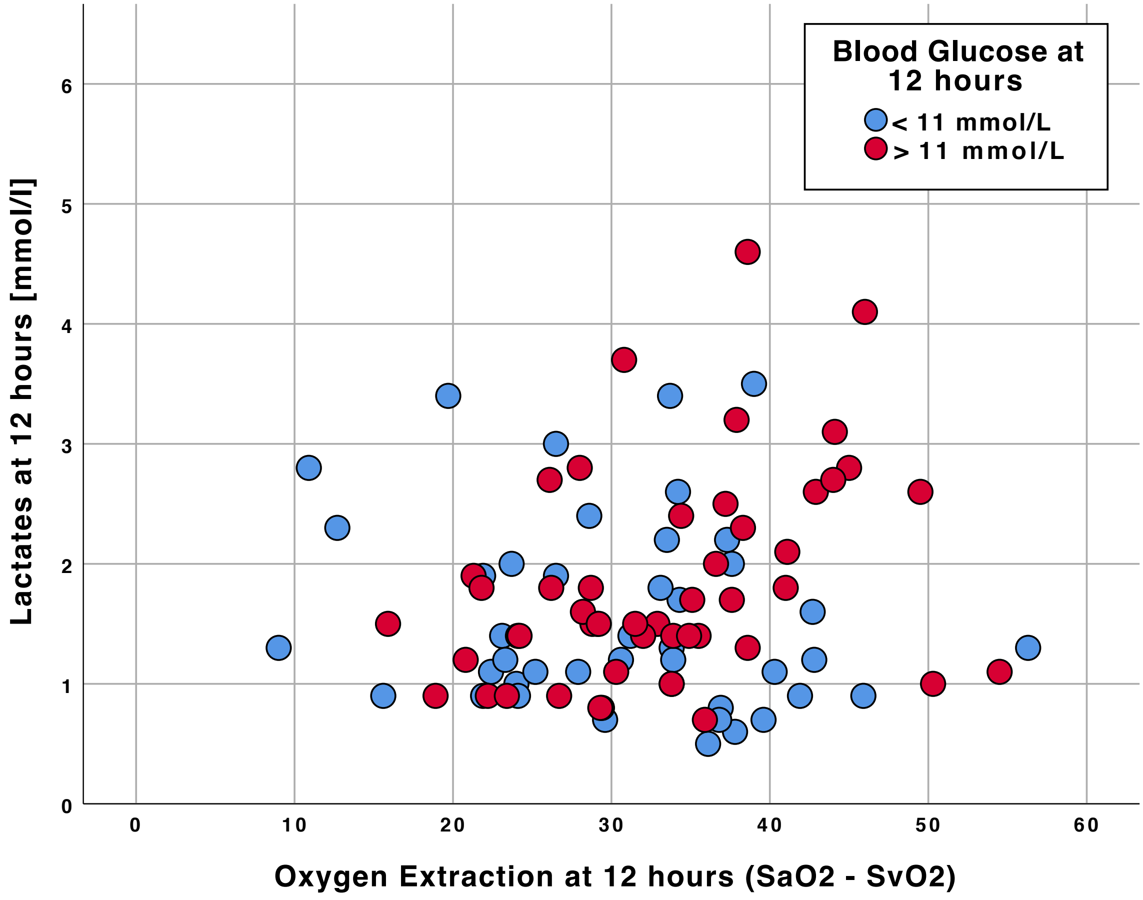

Figure S2: Oxygen delivery and lactate levels, at 12 hours, according to the blood glucose levels.

2B. Association between categorized variables, with two thresholds for Oxygen Extraction

	Oxygen extraction < 30%	Oxygen extraction ≥ 30%
**Lactate < 2 mmol/L**	31	33
**Lactate ≥ 2 mmol/L**	8	21

Chi Square = 3.56, p=0.06

3) Association between Lactate and Blood Glucose, at 12 hours

3A. Correlation between continuous variables:

Spearman correlation between Blood Glucose and Lactates: R=0.27, p=0.007

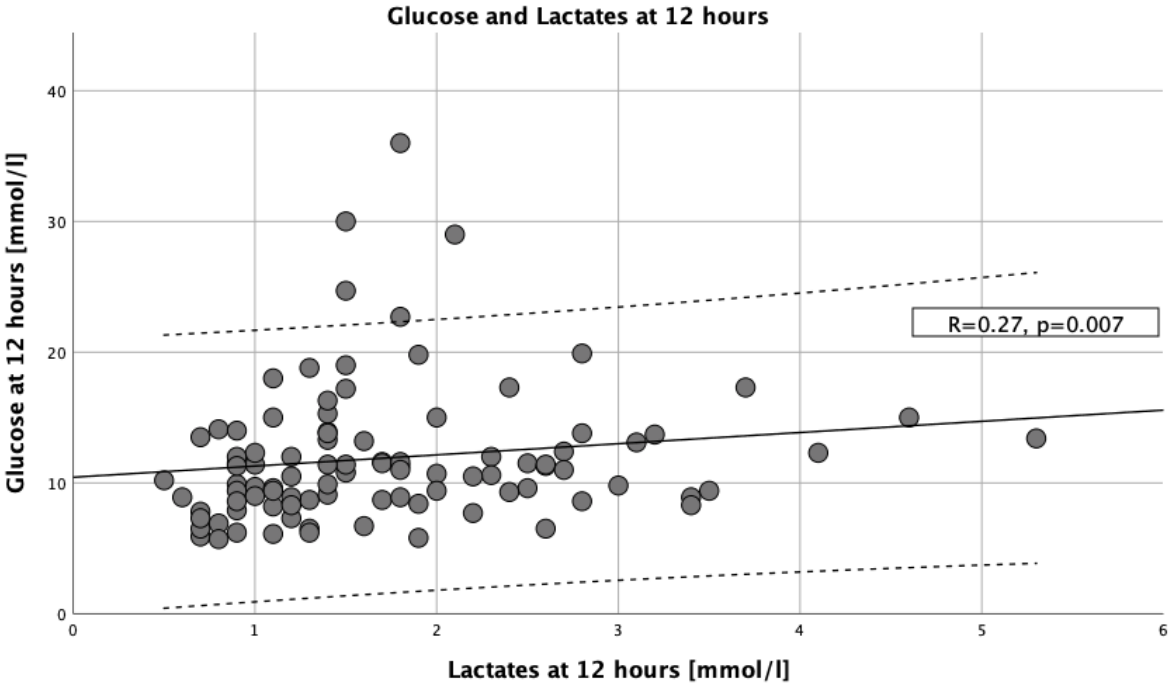

Figure S3: Correlation between lactate levels and Blood Glucose, at 12 hours, with 95% confidence interval.

3B. Association between categorized variables

	Blood Glucose < 11 mmol/L	Blood Glucose ≥ 11 mmol/L
**Lactate < 2 mmol/L**	33	31
**Lactate ≥ 2 mmol/L**	13	16

Chi Square = 0.36, p=0.55

4) Association between Blood Glucose, Lactate, and Oxygen extraction, at 12 hours

4A. Linear regression model with Lactate as outcome
Oxygen extraction: Beta=0.016 (95%CI -0.005 to 0.037), p=0.13
Blood Glucose: Beta=0.023 (95%CI -0.013 to 0.056), p=0.21

4B. Logistic regression model with Lactate > 2 mmol/L as outcome
Oxygen extraction: Exp(B)=1.04 (95%CI 0.99 to 1.10), p=0.10
Blood Glucose: Exp(B)=1.01 (95%CI 0.93 to 1.10), p=0.74

5) Description of patients who died

Supplemental Table: Description of patients who died

	Patient A	Patient B	Patient C
**Age [years]**	1.9	9.2	9.3
**Weight [kg]**	6.46	21.0	20.7
**RACHS-1***	3	2	3
**Lactate at 4 hours [mmol/L]**	2.0	2.9	2.9
**Blood Glucose at 4 hours [mmol/L]**	28	16.4	12.9
**Oxygen Extraction at 4 hours**	29%	24%	56%
**Lactate at 12 hours [mmol/L]**	2.1	1.1	2.6
**Blood Glucose at 12 hours [mmol/L]**	29.0	18.0	11.3
**Oxygen Extraction at 12 hours**	41%	30%	43%

*Exact diagnosis not presented, to ensure anonymity
